# Laparoscopic natural orifice specimen extraction colectomy versus conventional laparoscopic colorectal resection in patients with rectal endometriosis: a randomized, controlled trial

**DOI:** 10.1097/JS9.0000000000000728

**Published:** 2023-09-14

**Authors:** Noémi Dobó, Gabriella Márki, Gernot Hudelist, Noémi Csibi, Réka Brubel, Nándor Ács, Attila Bokor

**Affiliations:** a Department of Obstetrics and Gynecology, Semmelweis University; b MedEnd Institute, Budapest, Hungary; c Department of Gynecology, Center for Endometriosis, Hospital St. John of God, Rudolfinerhaus Private Clinic and Campus, Vienna, Austria

**Keywords:** colorectal endometriosis, endometriosis health profile 30, gastrointestinal quality of life index, low anterior resection syndrome, natural orifice specimen extraction

## Abstract

**Background:**

The conventional laparoscopic approach for the surgical management of deep endometriosis (DE) infiltrating the rectum appears to ensure improved digestive functional outcomes. The natural orifice specimen extraction (NOSE) technique for the treatment of colorectal DE can significantly accelerate postoperative recovery; however, data on gastrointestinal function following conventional laparoscopic segmental bowel resection (CLR) compared with NOSE colectomy (NC) for DE are sparse.

**Materials and methods:**

Between 30 September 2019 and 31 December 2020, a randomized, open-label, two-arm, parallel-group controlled trial with women aged 18–45 years was conducted at University Hospital.

Ninety-nine patients were randomized to CLR or NC, with DE infiltrating at least the muscular layer, at least 50% of the circumference of the bowel, up to 15 cm from the anal verge, exhibiting pain and bowel symptoms and/or infertility. The primary endpoint was bowel function, represented by low anterior resection syndrome (LARS). Secondary parameters included the Endometriosis Health Profile 30 (EHP30), Gastrointestinal Quality of Life Index (GIQLI), Visual Analog Scale (VAS) scores preoperatively and at set times (1 and 6 months, 1 year) following surgery.

**Results:**

No significant differences were observed in the postoperative LARS scores, VAS, EHP30, and GIQLI between the NC and CLR groups. LARS scores did not reveal significant differences 12 months postoperatively compared to the preoperative values in both groups (CLR group *P*=0.93 versus NC group, *P*=0.87). GIQLI scores were significantly improved 12 months after the operation compared with baseline values in the CLR group (*P*=0.002) and NC group (*P*=0.001). Pain symptoms and quality of life scores significantly improved 12 months postoperatively in both groups.

**Conclusions:**

NC is a feasible surgical approach for treating patients with rectal DE. Our study did not show a statistically significant difference between CLR and NC techniques in mid-term digestive and pain outcomes.

## Graphical abstract

**Figure:**
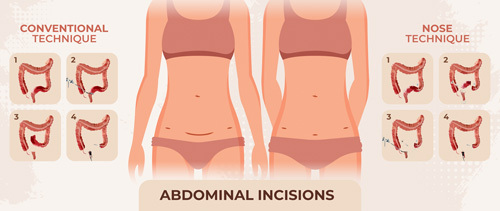

## Introduction

Highlights

- Both natural orifice specimen extraction colectomy and conventional laparoscopic colorectal resection equally improve the functional outcomes and the quality of life of patients with deep infiltrating rectal endometriosis.
- Pain symptoms and quality of life were significantly improved 12 months postoperatively compared to the preoperative values in both groups.
- Impaired bowel function alone should not be an indication for the surgical treatment of colorectal endometriosis.

Colorectal endometriosis is observed in up to 3–37% of women with a known diagnosis of endometriosis^1,2^, and is most commonly located in the rectum or sigmoid colon^3^. Although bowel endometriosis may be entirely asymptomatic, colorectal deep endometriosis (DE) commonly affects health-related quality of life^3–7^.

Laparoscopic surgery is the most widely accepted surgical approach in cases of bowel involvement^8–10^, but the optimal type of resection remains unresolved^11–14^.

Surgical specimen removal after segmental bowel resection can either be accomplished by minilaparotomy or by the natural orifice extraction technique^15–18^. According to previously published systematic reviews and meta-analyses, the natural orifice specimen extraction (NOSE) procedure is safe, may significantly reduce the duration of hospital stay, accelerate postoperative recovery with a better cosmetic outcome, results in lower postoperative pain, and complication rates^19–21^. Several studies have demonstrated a significant drop in pain scores and amelioration of impaired sexual functioning in women following the surgical resection of colorectal endometriosis; nevertheless, long-term, prospectively collected data on gastrointestinal well-being after segmental bowel resection for DE in a large cohort of patients are sparse^11,22–25^.

The primary objective of this study was to report the short-term and medium-term outcomes of bowel function as reflected by the Low Anterior Resection Syndrome (LARS) score^26^.

Our secondary outcomes assessed Visual Analog Scale (VAS) scores, Gastrointestinal Quality of Life Index (GIQLI)^27,28^, Endometriosis Health Profile 30 (EHP30)^29,30^, rate of complications, length of hospital stay, and recovery after NOSE versus conventional nerve and vessel spearing – colectomy for colorectal endometriosis.

## Material and methods

This study was designed in accordance with the Declaration of Helsinki and approved by the Institutional Ethical and Review Board of the University for the protection of human subjects (no.: 58723-4/2016/EKU) on 8 December 2016.

We conducted a single-center, randomized, open-label, two-arm, parallel-group controlled trial to assess functional outcomes and endometriosis-related pain changes in women undergoing NOSE colectomy (NC) or conventional laparoscopic resection (CLR) for the management of colorectal DE between 30 September 2019 and 31 December 2020, at University Hospital. The secondary outcomes included complication rates and fertility outcomes. The mean follow-up time was 14±2.6 months.

Inclusion criteria were being clinically diagnosed (by at least one imaging technique or via a previous surgery) as having intestinal deep infiltrating endometriosis up to 15 cm from the anus, involving at least the muscular layer in depth, and at least 50% of the recto-sigmoideal circumference in case of patients complaining of pain and/or infertility, and age 18–45 years. Ongoing pregnancy and suspected malignancy were excluded. Written informed consent was obtained from all patients before randomization.

All selected women underwent clinical examination by a gynecologist experienced in colorectal surgery for endometriosis, as well as a transvaginal ultrasound examination. Transvaginal sonography was performed to assess whether the rectum was involved, and to estimate the depth of rectal wall infiltration according to the IDEA protocol^31^. In case of parametrial involvement a pelvic MRI was also performed. All the surgeries were performed by the same surgical team.

**Figure 1:**
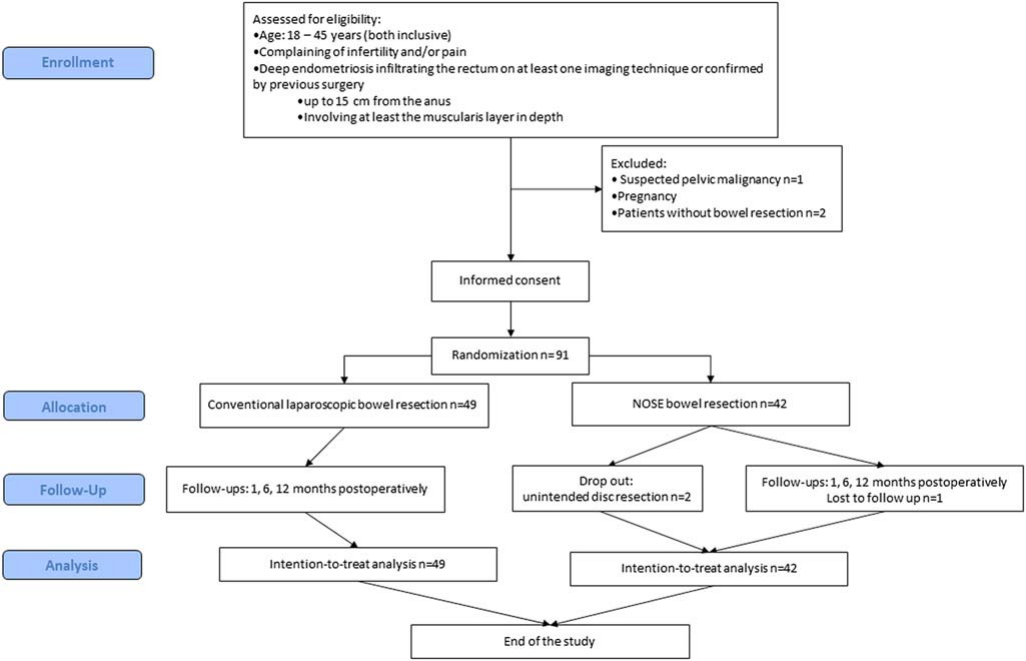
Flow diagram (CONSORT 2010) for study of patients, who underwent NOSE or conventional laparoscopic segmental resection for rectal DE.

This work has been reported in line with the Consolidated Standards of Reporting Trials (CONSORT, Supplemental Digital Content 1, http://links.lww.com/JS9/A991) Guidelines (Fig. 1)^32^.

## Theory/calculation

### Questionnaires

Patients were asked to complete baseline questionnaires including questions about pelvic organ function, pelvic pain, and quality of life related to endometriosis using the VAS (dysmenorrhea, dyspareunia, dyschezia, dysuria, and chronic pelvic pain), EHP30 (pain, control and powerlessness, emotional well-being, social support, self-image, sexuality)^29,30^, the GIQLI^27,28^, and the LARS^26^ score to assess bowel function preoperatively (T0), and at 30 days (T1), 6 months (T2), and 1 year (T3) postoperatively.

### Infertility assessement

The endometriosis fertility index^33^ was calculated for each procedure. The clinical pregnancy rates were calculated during the first postoperative year.

### Randomization

Assigning patients to NOSE or conventional colorectal resection was based on a randomization list using a simple randomization method. To determine the allocation sequence, computer-based coin flipping (http://www.random.org) was carried out by a staff member with no clinical involvement in the study. Randomization started after the patient had completed all baseline assessments and provided written consent to be enrolled in the trial. Patients were analyzed within the group to which they were allocated, irrespective of whether they had experienced the intended intervention (intention-to-treat analysis).

Blinding in our study was not feasible.

### Statistical analysis

The study data were evaluated using descriptive statistical methods such as average, median, range frequency, and distribution. Variables were tested for normality using the Kolmogorov–Smirnov–Lilliefors and the Shapiro–Wilk tests; skewness and kurtosis were also examined. Groups of values without a normal distribution were compared using the Mann–Whitney *U* test, Kruskal–Wallis test, Fisher’s exact test, or Wilcoxon signed-rank test. Where the distribution allowed, an independent samples *t*-test was used for continuous variables. The evolution of quality of life and digestive symptoms was tested based on the records at 12 months.

All tests were two-sided, and *P*<0.05 was accepted as a significant difference. Statistical analyses were performed using IBM SPSS version 17 (IBM Corp.).

### Sample size calculations

Based on the results of our previously published^22^, multicenter, retrospective study on the data of 205 patients with low rectal endometriosis undergoing laparoscopic surgery, we ascertained that in our center, the mean LARS scores were 29±12 in the CLR group and 21±9 in the NC group at one year postoperatively. In our present study, the randomized case selection enabled us to build a cohort where at least 80% statistical power was set as the target. LARS is the key characteristic/measure of a technique’s outcome, supporting the power calculation performed (https://clincalc.com/Stats/SampleSize.aspx) using our previous data. To detect low anterior resection syndrome using the LARS score as a continuous variable by assessing two independent study groups with a mean of 29±12 for the CLR group and 21±9 for the NC group, a sample size of 70 patients was required with 80% power at 0.05 alpha. Based on our previous experience, we predicted a drop-out/loss to follow-up rate of 22%; therefore, our final sample size was 91 patients.

### Surgical procedures

The primary objective of surgery was to achieve visibly complete elimination of all endometriotic lesions, regardless of the technique used. The team included two gynecological surgeons with extensive experience in endometriosis surgery. Nerve-sparing and vessel-sparing techniques were used to preserve the inferior hypogastric plexus, hypogastric nerves, and splanchnic nerves on at least one side.

All patients were placed in the modified dorsal lithotomy position. Pneumoperitoneum was induced by inserting a Veress needle (Karl Storz, Tuttlingen, Germany) into the umbilicus. A 4-port approach was used. Subsequently, the patient was placed in the steep Trendelenburg position. During the procedures, adhesiolysis was implemented for mobilization of the rectum and sigmoid colon in cases of pelvic adhesions. The ureters were dissected at the level of the uterine arteries. Limited tubular resection in a mesosparing manner close to the bowel was used to preserve the branches of the inferior hypogastric plexus.

During the complete intra-abdominal NOSE procedure, after skeletonization and isolation of the affected rectum, the rectum was tied off laparoscopically proximally and distally to the DE nodule with a nonabsorbable suture (Dafilon 0; B Braun AG). A laparoscopic atraumatic temporary intestinal clamp (Aesculap) was placed to decrease the chance of fecal spillage, cephalad to the resection line. A transverse colostomy was performed in healthy tissue using a harmonic scalpel to deliver the anvil from a circular stapler (Proximatew ILS CDH 29, Ethicon Endo-Surgery) introduced through the anus using a sterile laparoscopic camera sleeve (folded laparoscopic camera sleeve; 3M, St Paul). With the use of a camera sleeve for anvil introduction into the abdominal cavity, the possibility of peritoneal cavity contamination was reduced^18^.

A completely transected specimen was extracted transrectally through the camera sleeve in a specimen retrieval bag. The proximal part of the anastomosis was created by suturing the anvil in place with the purse-string of a monofilament laparoscopic suture (PDS 2.0; Ethicon, Inc.). The intestinal clamp was removed. The distal rectum was closed by using an endoscopic linear stapler. End-to-end anastomosis was performed by using a circular stapler.

With conventional segmental bowel resection, dissection was continued towards the pelvic floor distally to the affected segment. The rectum was then skeletonized using a vessel-sealing device (Harmonic Scalpel ACE; Ethicon Endo-Surgery) laterally and anteriorly, entering the rectovaginal septum and preserving the posterior wall of the vagina. The distal rectum was closed using an endoscopic linear stapler (Echelon Flex Endopath, Ethicon Endo-Surgery). The mobilized rectum with the specimen was retrieved through a small suprapubic incision. The anvil of a conventional circular stapler was introduced into the proximal colon following the placement of a purse-string suture (PDS 2.0; Ethicon, Inc.). Circular stapled colorectal end-to-end anastomosis was then performed (Proximatew ILS CDH 29).

At the end of both procedures, extensive saline irrigation was performed, and the integrity of the suture line at the distal rectum was verified using the Michelin test. A drain was conventionally left in place in the pouch of Douglas^18,22^.

The perioperative care was similar in patients from both arms of the study as the participants received the same medication and nursing technique.

### Outcomes

The primary outcome of our study was to test whether the NOSE colectomy technique offered any advantage in terms of functional outcomes and quality of life when compared to conventional laparoscopic bowel resection. As secondary outcomes, the complication rates, time to recovery, and impact on fertility of both procedures were assessed.

## Results

A total of 91 patients were enrolled in the study between 30 September 2019 and 31 December 2020, at the University Hospital, with 42 randomly assigned to the NC arm and 49 assigned to the CLR arm. One patient was lost to follow-up (Fig. 1).

The demographic and clinical characteristics of the participants are shown in Table 1. The mean age of the patients in the NC and CLR groups was 35±5 and 34±5 years, respectively. All the patients had one or more pain or intestinal symptoms. Twenty-six patients in the NC group (63.4%) and 35 patients in the CLR group (71.4%) had undergone one or more previous surgeries for endometriosis, excluding colorectal procedures.

**Table 1:** Demographic data.

Patients’ baseline characteristics	NOSE (*n*=42)	Conventional (*n*=49)
Age (years)	35±5	34±5
BMI (kg/m^2^)	21±3	23±4
ASA score		
I	29 (70.7%)	32 (65.3%)
II	12 (29.3%)	17 (34.7%)
Infertility	14 (34.1%)	20 (40.8%)
Previous surgeries for endometriosis		
NO surgery	15 (36.6%)	14 (28.6%)
1 surgery	17 (41.5%)	17 (34.7%)
2 or more surgeries	9 (21.9%)	18 (36.7%)
Type of previous surgeries		
No of patients with previous surgeries	26 (63.4%)	35 (71.4%)
Previous laparoscopy	23 (56.1%)	32 (65.3%)
Previous laparotomy	7 (17.1%)	12 (24.4%)
Previous pregnancy/delivery		
Pregnancy	12 (29.3%)	12 (24.4%)
Delivery	9 (21.9%)	5 (10.2%)

Data are *n* (%) and mean±SD.

ASA, American Society of Anaesthesiologists score; NOSE, Natural Orifice Specimen Extraction.

Tables 2 and 3 present intraoperative findings and postoperative complications, respectively. The anatomical distribution of the endometriotic DE lesion sites was similar in both groups. There was no difference in the length of hospital stay between the NC and CLR groups (5,3±3 days in the NC group versus 5,7±2 days in the CLR group). All cases were confirmed by histological examination.

**Table 2:** Intraoperative findings.

	NOSE (*n*=42)	Conventional (*n*=49)	*P*
Operative time (min)	139±97	147±76	0.7
Hospital stay (day)	5.3±3	5.7±2	0.2
Blood loss (ml)	22±16	24±22	0.4
Localization of deep nodules of the digestive tract			
rectum	34 (82.9%)	42 (85.7%)	0.3
sigmoid/rectum junction	5 (12.2%)	7 (14.3%)	0.5
sigmoid	9 (21.9%)	7 (14.3%)	0.09
sigmoid and appendix	2 (4.8%)	2 (4.0%)	0.6
ileum	0 (0.0%)	6 (12.2%)	0.03
coecum	2 (4.8%)	6 (12.2%)	0.2
Segmental resection of ileum/coecum	2 (4.8%)	12 (24.4%)	0.7
Appendectomy	2 (4.8%)	2 (4.0%)	0.1
Omental/Mesorectal flap	3 (7.3%)	2 (4.0%)	0.9
Protective colostomy	0 (0.0%)	1 (2.0%)	0.6
Diameter of largest rectal nodule (mm)	27±3.8	29±2.5	0.5
Deepest infiltration of the rectum			
mucosa	5 (12.2%)	2 (4.0%)	0.5
submucosa	8 (19.5%)	6 (12.2%)	0.9
muscularis	28 (68.3%)	41 (83.8%)	0.2
Height of the lowest nodule (from the anal verge)			
below 7cm	19 (46.3%)	20 (40.8%)	0.8
above 7cm	22 (53.7%)	29 (59.2%)	0.6
Length of the removed bowel segment (mm)	67±2.7	83±3.8	0.06
ASRM score	51±24	47±17	0.8
ENZIAN			
Compartment A	30 (73.2%)	43 (87.7%)	0.1
Compartment B	15 (36.6%)	12 (24.4%)	0.9
Compartment C	42 (100%)	49 (100%)	n.a
Compartment FA	27 (65.9%)	25 (51.0%)	0.8
Compartment FB	11 (26.8%)	10 (20.4%)	0.3
Compartment FU	0 (0.0%)	7 (14.3%)	0.02
Compartment FI	13 (31.7%)	21 (42.9%)	0.7
Compartment FO	1 (2.4%)	3 (6.1%)	0.9
Concomitant management of			
ovarian endometrioma	12 (29.3%)	15 (30.6%)	0.5
bladder nodule	11 (26.8%)	10 (20.4%)	0.3
rectovaginal space DE	30 (73.2%)	43 (87.7%)	0.2
vaginal infiltration	14 (34.1%)	19 (38.7%)	0.4
peritoneal disease	39 (95.1%)	47 (95.9%)	0.3
ureteral DE	0 (0.0%)	7 (14.3%)	0.02
EFI score	4.8±2.1	5.2±1.8	0.3
Patency of fallopian tubes			
Unilateral occlusion	4 (9.8%)	15 (30.6%)	0.007
Bilateral occlusion	20 (48.7%)	18 (36.7%)	0.2
Patent	17 (41.5%)	16 (32.7%)	0.1

Data are *n*(%) and mean±SD.

ASRM, American Society of Reproductive Medicine; DE, deep endometriosis; EFI, Endometriosis Fertility Index; NOSE, Natural Orifice Specimen Extraction.

**Table 3:** Postoperative surgical complications.

	NOSE (*n*=42)	Conventional (*n*=49)	*P*
Complications according to Clavien–Dindo classification			
I			
Bladder atony (max 7 days)	5 (11.9%)	4 (8.1%)	0.739
Fever	4 (9.52%)	3 (6.1%)	0.705
Clostridium difficile infection	4 (9.52%)	3 (6.1%)	0.705
II			
Rectal bleeding	2 (4.76%)	2 (4.1%)	n.a
Ileus	0 (0.0%)	1 (2.0%)	n.a
III			
Anastomotic leakage	1 (2.38%)	0 (0.0%)	n.a
Rectovaginal fistula	0 (0.0%)	1 (2.0%)	n.a
IV	0 (0.0%)	0 (0.0%)	n.a.

Data are *n*(%).

NOSE, Natural Orifice Specimen Extraction.

Fisher Exact test has been executed.

The intraoperative classification of endometriosis was performed according to the rASRM and ENZIAN classification systems during all procedures^34,35^, see Table 2.

When comparing the NC and CLR groups with regard to the rates of grade I and II postoperative complications, no statistically significant difference was found. According to the Clavien–Dindo classification, we observed two severe (grade III or higher) complications (2.27%): one anastomotic leakage in the NC group and one rectovaginal fistula in the CLR group^36^. These were managed with a covering ileostoma repair. Intestinal continuity was restored within three months.

Table 4 shows preinterventional and postinterventional values of GIQLI, EHP30, LARS, and VAS scores, which were comparable between the two arms. The LARS scores did not reveal significant differences 12 months after the operation compared to the baseline values in both groups (NC: T0=22.2±11.7, T3=16.33±14.18, *P*=0.87; CLR: T0=21.41±10.2, T3=17.90±11.1, *P*=0.934). GIQLI significantly improved 12 months after the operation compared with the baseline values in both groups NC: T0=96.17±16.07, T3=112.13±16.8, *P*=0.001 and CLR: T0=95.44±23.1, T3=111.39±18.4, *P*=0.002.

**Table 4:** Preoperative and postoperative assessment of digestive function and quality of life.

	NOSE (*n*=42)					Conventional (*n*=49)				
	T0		T3			T0		T3		
Questionnaires	Mean	SD	Mean	SD	*P*	Mean	SD	Mean	SD	*P*
LARS	22.2	11.7	16.33	14.18	0.87	21.41	10.2	17.9	11.18	0.934
GIQLI	96.17	16.07	112.13	16.8	0.001	95.44	23.11	111.39	18.48	0.002
EHP30										
Pain	31.7	26.3	6	9.2	0.001	30.8	25.5	10.2	14.2	0.001
Emotional well-being	43.4	23.4	17.4	15.8	0.001	38.9	25.5	16.6	17.7	0.01
Control and powerlessness	45.9	26.5	11.4	16.2	0.016	41.1	31.9	12.2	16.5	0.014
Self-Image	37	26.5	12.6	15	0.001	37	31.8	18	23.9	0.000
Social suppose	24.3	24.6	5.1	9.1	0.000	31.6	31	6.9	14.4	0.000
Sexuality	36.1	34.9	12.3	20.5	0.043	43.9	34.5	19.1	25.3	0.003
VAS scores										
Dysmenorrhea	4	1.9	3	2.1	0.03	5	5.1	4	3.1	0.24
Chronic pelvic pain	6	5.2	3	2.2	0.004	7	3.3	2	2.3	0.0001
Dyspareunia	6	4.2	3	3.1	0.0001	6	4.5	3	2.5	0.0001
Dysuria	3	5.8	1	1.7	0.04	2	5.8	1	2.8	0.27
Dyschezia	6	4.4	3	2.1	0.0003	5	4.7	2	1.7	0.0001

EHP30, Endometriosis Health Profile 30; GIQLI, Gastrointestinal Quality of Life Index; LARS, Low Anterior Resection Syndrome; NOSE, Natural Orifice Specimen Extraction; T0, preoperative; T3, 12 months after surgery; VAS, Visual Analogue Scale.

Mann–Whitney *U* test was used.

The EHP30 scores significantly improved 12 months after the operation compared to the preoperative values in both groups. The overall GIQLI, EHP30, and LARS scores did not reveal significant differences between the two arms 12 months after surgery.

**Figure 2:**
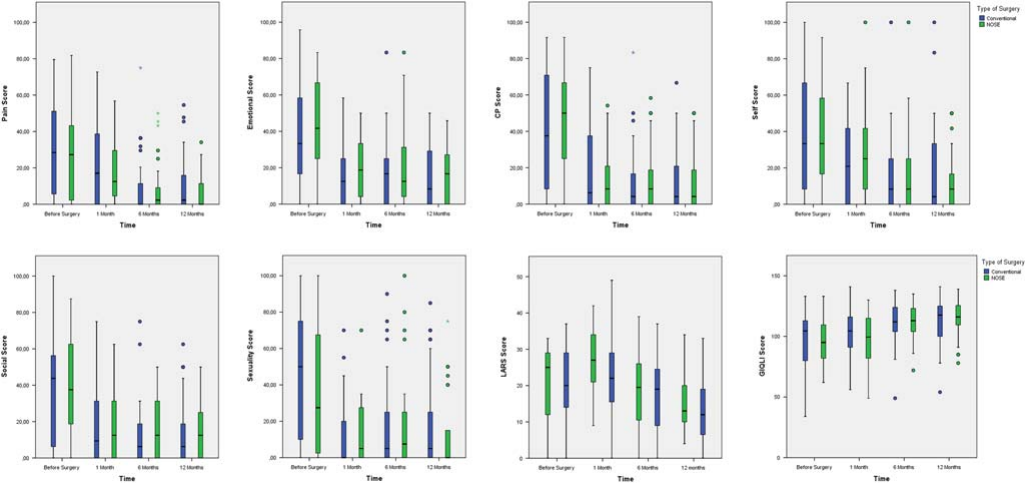
Endometriosis Health Profile 30 scales (pain, emotional well-being, control and powerlessness-CP, self-image, social support, sexuality), LARS (Low Anterior Resection Syndrome) score and GIQLI (Gastrointestinal Quality of life Index) score in conventional and NOSE surgery groups preoperatively, 1 month, 6 months, and 1 year after the surgery.

Figure 2 presents preoperative, and postoperative EHP30 scores, GIQLI and LARS scores.

Regarding pain symptoms, the chronic pelvic pain, dyspareunia, and dyschezia VAS scores were significantly improved in both groups compared with the baseline scores 1 year after surgery (chronic pelvic pain: NC T0=6±5.2, T3=3±2.2, *P*=0.004; CLR: T0=7±3.3, T3=2±2.3, *P*=0.0001; dyspareunia: NC T0=6±4.2, T3=3±3.1; *P*=0.0001; CLR: T0=6±4.5, T3=3±2.5, *P*=0.0001; dyschezia: NC T0=6±4.4, T3=3±2.1, *P*=0.0003; CLR: T0=5±4.7, T3=2±1.7, *P*=0.0001). We found statistically significant decreases in the intensity of dysmenorrhea (T0=4±1.9, T3=3±2.1, *P*=0.03) and dysuria (T0=3±5.8, T3=1±1.7, *P*=0.04) after 1 year of follow-up in the NC group.

There was no statistically significant difference in the change in LARS, GIQLI, EHP30, and VAS scores between the NOSE and conventional treatment groups 12 months after surgery when compared to preoperative values (T3 minus baseline) see Table 5.

**Table 5:** Clinical assessment 12 months after surgery.

	NOSE (*n*=42)	Conventional (*n*=49)	*P*
LARS delta T3-T0	−5.93±10.58	−6.80±11.50	0.923
Min–max	−24–18	−34–16	
GIQLI delta T3-T0	18.03±19.51	15.07±20.75	0.654
Min–max	−10–70	−27–70	
EHP30 delta T3-T0			
Pain	−23.01±23.85	−21.21±22.18	0.666
Min–max	−70.45–25	−63.64–18.18	
Emotional well-being	−26.21±24.08	−23.00±24.97	0.356
Min–max	−70.83-41.67	−75-29.17	
Control and powerlessness	−34.35±26.79	−29.77±27.89	0.375
Min–max	−91.67-25	−87.50-29.17	
Sexuality	−21.40±28.49	−29.90±31.75	0.900
Min–max	−95-40	−90–60	
Self-Image	−24.18±26.07	−18.75±26.77	0.311
Min–max	−91.67–8.33	−90–60	
Social support	−20.79±25.56	−22.02±23.17	0.865
Min–max	−75–30	−70–10	
VAS scores delta T3-T0			
Dysmenorrhea	−2.41±3.94	−3.25±4.37	0.510
Min–max	−9.65-6.78	−10-6.21	
Chronic pelvic pain	−4.29±3.50	−3.27±4.10	0.331
Min–max	−10–3.36	−10–7.11	
Dyspareunia	−3.79±3.45	−3.42±3.91	0.903
Min–max	−10-17	−10-8.3	
Dysuria	−0.52±1.58	−0.16±2.04	0.270
Min–max	−8–1.73	−6–8.08	
Dyschezia	−2.99±3.54	−3.60±4.27	0.281
Min–max	−10–4.90	−10–7.75	

EHP30, Endometriosis Health Profile 30; GIQLI, Gastrointestinal Quality of Life Index; LARS, Low Anterior Resection Syndrome; NOSE, Natural Orifice Specimen Extraction; T0, preoperative; T3, 12 months after surgery; VAS, Visual Analog Scale.

Mann–Whitney *U* test was executed.

The results of follow-up assessments for each time point (T0, T1, T2, and T3) are depicted in Supplementary Table 1 (Supplemental Digital Content 2, http://links.lww.com/JS9/A992).

During the 14±2.6 months of follow-up, 22 patients with active child wishes achieved pregnancy: 8 in the NC arm (21%) and 14 in the CLR arm (29%) (*P*=0.867). Among them, two (4%) and two (5%) conceived spontaneously. Seven (NC group, 18%) and seven (CLR group, 14%) live births were reported in both groups.

## Discussion

To the best of our knowledge, this is the first prospective randomized study on bowel function, quality of life, and pain outcomes of two different specimen extraction techniques for the surgical treatment of recto-sigmoideal DE.

We observed no statistically significant difference in the occurrence of LARS and GIQLI scores in our cohort of patients who underwent either the NOSE-technique or the conventional nerve and vessel-sparing bowel resection after one year of follow-up. We found no evidence confirming the superiority of the NOSE-technique technique over conventional laparoscopic segmental resection in terms of bowel function and quality of life.

The complex of symptoms consisting of incontinence due to flatus and/or feces, constipation, and frequent bowel movements is referred to as LARS. However, little is known about the exact cause of LARS. Several studies have addressed the symptoms of LARS, but significant variability exists in the reporting of outcomes after anterior resection^37^.

Riiskjaer *et al*.^25^, in their prospective observational study, reported a significant increase in the frequency of defecation one year after surgery, probably as a result of decreased reservoir capacity. The mean overall LARS score was not significantly different 1 year after surgery. Most patients had minor/major LARS, both before and after surgery. Our study assessed the occurrence of LARS both preoperatively and postoperatively, and found no statistical difference between the extent of LARS before and after surgery in the investigated cohort of women.

In the prospective cohort trial by Hudelist *et al*.^11^ comparing segmental and disk resection, they did not observe significant differences in long‐term functional outcomes regarding minor or major LARS (*P*=0.48 and *P*=0.66, respectively). These findings are in line with those previously reported by our group^22^. In agreement with recent findings^13,25^, we would like to emphasize that impaired bowel function alone should not be an indication for bowel surgery.

In the present study, GIQLI scores were comparable in both groups and showed a statistically significant improvement over time from the baseline values until 1 year after surgery in the NC group (*P*=0.001) and CLR group (*P*=0.002) groups. This is consistent with Roman *et al*.^13^, who observed improved and comparable GIQLI scores one year after anterior segmental resection compared to disk excision.

Similar to previously published data^11,13,22,38,39^ our results confirm that radical excision of colorectal DE improves the quality of life and lowers pain scores.

As secondary outcomes, we assessed the occurrence of major surgical complications, time to recovery, and length of hospitalization. Our data correlate with previously reported data^18,39^; however, we could not confirm the shorter hospital stay and lower postoperative pain scores after NOSE colectomy in our cohort of patients.

Recently, a meta-analysis by Liu *et al*.^40^ reported the incidence of anastomotic leakage for the NOSE group was 3.6 compared to 5% in the conventional laparoscopic group. Another meta-analysis by Ma *et al*.^20^ showed that laparoscopic resection with NOSE resulted in fewer postoperative complications. From the pooled data of the two meta-analyses, the incidence of anastomotic leakage was not significantly different between the two groups. Thus, we conclude that laparoscopic colorectal surgery with NOSE is as safe as conventional laparoscopic surgery. Previous studies have reported faster gastrointestinal recovery, less postoperative pain, and a shorter hospital stays following laparoscopic colorectal surgery with NOSE^18,19,41^. The results of two recently published meta-analyses also suggested that the NOSE group had less postoperative pain and shorter hospital stay than the conventional laparoscopic anterior resection group^20,21,40^. In the aforementioned meta-analyses, the use of the NOSE-technique was clearly associated with a shorter hospital stay, less postoperative pain, and fewer perioperative complications, although this was not confirmed in our study.

In agreement with recent findings^13,25^ we would like to underline the fact that impaired bowel function alone should not be an indication for bowel surgery since we noted the presence of different degrees of LARS in the majority of our patients preoperatively and the LARS scores did not significantly change after surgery.

Keane *et al*. recently defined LARS using a robust methodology that includes multiple stakeholders. This innovative approach suggested that both symptoms and consequences are important priorities in LARS^42^. These important priorities may lead to better identification of patients who experience bowel dysfunction and offer a better perception of LARS in the future. Moreover, further research is needed to elucidate the underlying mechanism of the presence of LARS before colorectal resection.

In the present study, the difference between specimen extraction techniques after colorectal resection for DE had no effect on the functional/surgical outcome or quality of life.

The wider implication of our study is that NOSE colectomy offers the same benefits regarding bowel function, amelioration of pain symptoms, and quality of life as conventional laparoscopic anterior resection. Furthermore, after both procedures, the occurrence of LARS was lower than that previously reported^23,25^, and was similar to our recently published multicentric data^22^.

As all surgeries were performed by the same surgical team, it is less likely that unbalanced patient enrollment significantly affected the outcomes.

An obvious study limitation was our lack of blinding; however, this was not feasible because of the clinical nature of our study. The simple randomization method used in our study represents another limitation, because it resulted in unequal study groups.

We report no imprecision in patient selection or detection, but a potential source of attrition bias in our data occurred in one case (loss to follow-up) exclusively in the NOSE group.

## Conclusion

Our data demonstrated that both NOSE and conventional laparoscopic colectomy are safe methods for the surgical treatment of colorectal DE. The occurrence of long-term bowel dysfunction does not appear to be related to a specific surgical technique. The external validity of our outcomes must be investigated in multicenter prospective randomized trials in a larger cohort of patients.

## Ethical approval

This study was approved by the Institutional Ethical and Review Board of Semmelweis University for the protection of human subjects (no.: 58723-4/2016/EKU) on 8 December 2016.

## Consent

Written informed consent was obtained from the patient for publication and any accompanying images. A copy of the written consent is available for review by the Editor-in-Chief of this journal on request.

## Sources of funding

None.

## Author contribution

N.D.: study concept and design, acquisition of data, analysis and interpretation of data, manuscript drafting, and critical discussion; G.M.: study concept and design, analysis and interpretation of data, manuscript drafting, and critical discussion; N.C.S., R.B.: acquisition of data, manuscript drafting, and critical discussion; G.H: analysis and interpretation of data, manuscript drafting, and critical discussion; N.Á.: manuscript drafting and critical discussion; A.B.: study concept and design, acquisition of data, analysis and interpretation of data, manuscript drafting, and critical discussion.

## Conflicts of interest disclosure

All authors declare that no conflict of interest or financial ties to disclosure.

## Research registration unique identifying number (UIN)

ClinicalTrials.gov ID:NCT04109378. https://clinicaltrials.gov/ct2/show/NCT04109378?term=NCT04109378&draw=2&rank=1.

## Guarantor

Noémi Dobó MD., Attila Bokor MD, PhD, Msc.

## Data availability statement

Laparoscopic natural orifice specimen extraction (NOSE) colectomy versus conventional laparoscopic colorectal resection in patients with rectal endometriosis: a prospective randomized trial.

## Provenance and peer review

Not commissioned, externally peer-reviewed.

## Financial support and sponsorship

None.

## Presentation

The study was partially presented at ESHRE 38th Annual Meeting; on 3–6 July 2022, Milan, Italy.

## Supplementary Material

SUPPLEMENTARY MATERIAL
