# Supplementary material for: Laparoscopic natural orifice specimen extraction colectomy versus conventional laparoscopic colorectal resection in patients with rectal endometriosis: a randomized, controlled trial
Source: Int J Surg. 2023 Sep 14;109(12):4018–26. doi: 10.1097/JS9.0000000000000728 (PMC10720833; doi:10.1097/JS9.0000000000000728)
Supplement: SUPPLEMENTARY MATERIAL [file js9-109-4018-s002.docx]

| **Supplementary Table 1** | | | | | | | | | | |  |  |  |  |  |  |  |  |  |  |
| --- | --- | --- | --- | --- | --- | --- | --- | --- | --- | --- | --- | --- | --- | --- | --- | --- | --- | --- | --- | --- |
| **Pre- and postoperative assessment of functional and quality of life outcomes in each timepoint** | | | | | | | | | | |  |  |  |  |  |  |  |  |  |  |
| **Questionnaires** | **NOSE (n=42)** | | | | | | | | **Conventional (n=49)** | | | | | | | | **NOSE vs Conventional** | | | |
|  | **T0** | | **T1** | | **T2** | | **T3** | | **T0** | | **T1** | | **T2** | | **T3** | | **T0 vs T0** | **T1 vs T1** | **T2 vs T2** | **T3 vs T3** |
|  | **Mean** | **SD** | **Mean** | **SD** | **Mean** | **SD** | **Mean** | **SD** | **Mean** | **SD** | **Mean** | **SD** | **Mean** | **SD** | **Mean** | **SD** | **p** | **p** | **p** | **p** |
| **LARS** | 22.2 | 11.7 | 26.36 | 9.09 | 17.79 | 9.66 | 16.33 | 14.18 | 21.41 | 10.2 | 21.88 | 11.30 | 17.12 | 11.45 | 17.9 | 11.18 | 0.799 | 0.051 | 0.826 | 0.339 |
|  |  |  |  |  |  |  |  |  |  |  |  |  |  |  |  |  |  |  |  |  |
| **GIQLI** | 96.17 | 16.07 | 99.88 | 19.28 | 111.44 | 14.64 | 112.13 | 16.8 | 95.44 | 23.11 | 102.06 | 19.77 | 110.76 | 16.78 | 111.39 | 18.48 | 0.914 | 0.776 | 0.960 | 0.473 |
|  |  |  |  |  |  |  |  |  |  |  |  |  |  |  |  |  |  |  |  |  |
| **EHP 30** |  |  |  |  |  |  |  |  |  |  |  |  |  |  |  |  |  |  |  |  |
| ***Pain*** | 31.7 | 26.3 | 17.95 | 16.48 | 8.31 | 13.88 | 6 | 9.2 | 30.8 | 25.5 | 21.52 | 19.59 | 7.88 | 14.83 | 10.2 | 14.2 | 0.678 | 0.575 | 0.398 | 0.150 |
| ***Emotional well-being*** | 43.4 | 23.4 | 19.79 | 15.35 | 19.00 | 20.09 | 17.4 | 15.8 | 38.9 | 25.5 | 16.58 | 15.06 | 17.60 | 18.29 | 16.6 | 17.7 | 0.389 | 0.286 | 0.803 | 0.925 |
| ***Control and powerlessness*** | 45.9 | 26.5 | 15.21 | 17.44 | 13.01 | 16.65 | 11.4 | 16.2 | 41.1 | 31.9 | 18.20 | 21.00 | 10.54 | 16.30 | 12.2 | 16.5 | 0.552 | 0.771 | 0.384 | 0.767 |
| ***Self Image*** | 37 | 26.5 | 26.46 | 23.86 | 16.67 | 21.41 | 12.6 | 15 | 37 | 31.8 | 22.96 | 21.21 | 14.80 | 20.29 | 18 | 23.9 | 0.763 | 0.487 | 0.519 | 0.845 |
| ***Social support*** | 24.3 | 24.6 | 19.48 | 24.43 | 7.16 | 12.28 | 5.1 | 9.1 | 31.6 | 31 | 22.64 | 29.41 | 8.21 | 17.28 | 6.9 | 14.4 | 0.431 | 0.924 | 0.952 | 0.971 |
| ***Sexuality*** | 36.1 | 34.9 | 13.33 | 18.39 | 17.42 | 26.07 | 12.3 | 20.5 | 43.9 | 34.5 | 12.40 | 20.01 | 17.78 | 23.10 | 19.1 | 25.3 | 0.353 | 0.547 | 0.590 | 0.195 |
| **VAS scores** |  |  |  |  |  |  |  |  |  |  |  |  |  |  |  |  |  |  |  |  |
| ***Dysmenorrhea*** | 4 | 1.9 | 3 | 2.8 | 2 | 2.8 | 3 | 2.1 | 5 | 5.1 | 3 | 3.3 | 1 | 2.2 | 4 | 3.1 | 0.455 | 0.471 | 0.374 | 0.777 |
| ***Chronic pelvic pain*** | 6 | 5.2 | 2 | 3.1 | 2 | 2.4 | 3 | 2.2 | 7 | 3.3 | 3 | 3.0 | 2 | 2.7 | 2 | 2.3 | 0.791 | 0.702 | 0.912 | 0.286 |
| ***Dyspareunia*** | 6 | 4.2 | 1 | 1.9 | 1 | 2.6 | 3 | 3.1 | 6 | 4.5 | 1 | 1.4 | 1 | 1.9 | 3 | 2.5 | 0.851 | 0.188 | 0.766 | 0.612 |
| ***Dysuria*** | 3 | 5.8 | 2 | 2.4 | 1 | 1.2 | 1 | 1.7 | 2 | 5.8 | 2 | 3.0 | 1 | 1.3 | 1 | 2.8 | 0.700 | 0.950 | 0.964 | 0.443 |
| ***Dyschezia*** | 6 | 4.4 | 2 | 2.4 | 1 | 2.4 | 3 | 2.1 | 5 | 4.7 | 2 | 3.3 | 1 | 2.7 | 2 | 1.7 | 0.167 | 0.466 | 0.607 | 0.473 |
| **NOSE: Natural Orifice Specimen Extraction** | | | | | | | | | | | | | | | |  |  |  |  |  |
| **T0: preoperative T1: 1 month after surgery; T2: 6 months after surgery; T3: 12 months after surgery** | | | | | | | | | | | | | | | |  |  |  |  |  |
| **LARS: Low Anterior Resection Syndrome; GIQLI: Gastrointestinal Quality of Life Index** | | | | | | | | | | | | | | | |  |  |  |  |  |
| **EHP30: Endometriosis Health Profile 30** | | | | | | | | |  |  |  |  |  |  |  |  |  |  |  |  |
| **VAS: Visual Analoge Scale** | | | | | | | | |  |  |  |  |  |  |  |  |  |  |  |  |
| **Mann-Whitney U test was used.** | | |  |  |  |  |  |  |  |  |  |  |  |  |  |  |  |  |  |  |
